# Landscape of Post-Marketing Requirements Under the Pediatric Research Equity Act for Antibiotics from 2009–2024

**DOI:** 10.3390/antibiotics14060583

**Published:** 2025-06-06

**Authors:** Daniel Selig, Funmi Aminu, Sue Cammarata, Ting Chen, Lauren Dolak, Stephen Duprez, Stephanie Ecker, Lisa Gault, Sandra George, Margaret Harkins, Clayton Litchmore, Michael Serenko, William Waverczak, Doug Girgenti

**Affiliations:** 1 Melinta Therapeutics, LLC, 389 Interpace Pkwy, Suite 450, Parsippany, NJ 07054, USA; 2 Tunnell, 1235 Westlake Drive, Suite 280, Berwyn, PA 19312, USA

**Keywords:** anti-bacterial agents, antibiotics, clinical trials, drug development, infectious disease medicine, pediatrics, pharmaceutical industry, post-marketing commitments, PMR, PREA

## Abstract

**Background/Objectives:** We reviewed Post-Marketing Requirements (PMRs) under the Pediatric Research Equity Act (PREA) for antibiotics approved in adults from 2009 to 2024 to better understand factors associated with PMR study completion. **Methods**: Initial PMRs, including study design and completion timelines were extracted from Food and Drug Administration (FDA) approval letters. Studies were cross-referenced at clinicaltrials.gov, with follow-up from adult approval to study completion or through 31 December 2024. **Results**: Eighteen antibiotics were approved in adults from 2009 to 2024, with 53 associated PREA PMRs. A total of nine PMRs were excluded from analysis (six exclusions for projected study completion dates on or after 12/31/2024, one exclusion due to lack of information, and two exclusions because the study type was not categorizable as Phase 1 or Phase 2). Of the 44 remaining PMRs in the analysis set, the median pediatric study follow-up time from adult approval was 5.3 years (range 0.94 to 11.5 years), with a study completion rate of 54.5% (N = 24). Small- and medium-sized companies had a study completion rate of 10% (N = 2/20) over a median of 6.44 years of follow-up, with no pediatric approvals. Large pharmaceutical corporations had a significantly higher study completion rate of 91.6% (N = 22/24; adjusted hazard ratio 20.3 95%CI, 5.02 to 82.4) over a median follow-up time of 4.7 years and achieved pediatric approval with labelling updates for 75% of antibiotics (N = 6/8). **Conclusions**: Compared to larger organizations, smaller pharmaceutical companies have experienced difficulty in PREA PMR antibiotic study completion, which may be related to financial difficulties in the challenging market for antibiotics. To improve PMR study completion, smaller companies require continued financial support and innovation in study design. For pediatric antibiotic development, the FDA accepts the extrapolation of efficacy from well-conducted randomized adult trials (i.e., pharmacokinetics (PK) and the safety approach). Therefore, sponsors should consider the use of single-arm, non-comparative PK and safety study designs to reduce the size and scope of trials. Sponsors should also assess whether the evaluation of an antibiotic is necessary in adolescents, or if data in a surrogate population of adults (e.g., low-weight adults) may serve as adequate evidence for adolescent approval.

## 1. Introduction

The Pediatric Research Equity Act (PREA) is an outgrowth of legislation to address the recognized need for a strong ethical, scientific, and regulatory framework to conduct pediatric clinical drug trials [1,2]. Under PREA, the FDA has authority to require pediatric studies for several reasons, including products submitted under a New Drug Application (NDA), products submitted for a new formulation, or products submitted for a new indication. Successes associated with PREA include an increased number of pediatric clinical trials and improved pediatric drug labeling, both working towards a reduction of off-label use of adult medicines in children [3,4,5].

Despite successes associated with PREA, challenges in the conduct and operation of pediatric clinical trials remain. These challenges include the determination of the best study design, sample size, optimal study endpoints, and overcoming difficulty with enrollment [6,7,8], contributing to a low PREA-associated study completion rate [3]. An additional challenge specific to antibiotic development is a difficult financial market. In the post-marketing setting, the expected total 5-year cost after the post-market launch of an antibiotic is $242–622 million, with pediatric pharmacokinetic and safety studies accounting for $25–75 million of the total expenses. In comparison, revenue from antibiotic sales for the first nine quarters after launch ranges from approximately $0–80 million [9]. For small- or medium-sized companies relying on revenue from a single or multiple antibiotic products, total product revenue may be insufficient to cover 5-year post-market launch costs, resulting in delay or difficulty in completing pediatric studies. This is particularly important as the landscape of antibiotic development has shifted from large pharmaceutical companies to smaller biotech [10].

Recognizing many of these challenges, the FDA allows for an extrapolation approach to pediatric drug development where, “if the course of the disease and the effects of the drug are sufficiently similar in adult and pediatric patients, FDA may determine that pediatric effectiveness can be extrapolated from adequate and well-controlled studies in adult subjects” [7]. Additional FDA and International Council for Harmonisation of Technical Requirements for Pharmaceuticals for Human Use (ICH) guidance provides further detail on the extrapolation approach for pediatric antibiotic development. Guidance includes the following provisions: (i) allowing primary endpoints to be based on exposure matching (i.e., pharmacokinetics and safety); (ii) allowing the use of single-arm, non-comparator trials to determine the primary exposure matching endpoints; and (iii) noting that a dedicated PK study may not be necessary in all pediatric age groups. For example, an antibiotic study in a surrogate population of low-weight adults may be sufficient in lieu of a dedicated study in adolescents [11,12,13].

Given the challenges in completing pediatric studies mandated under PREA, the difficult financial market for antibiotics, and the shift of antibiotic development from large pharmaceutical companies to smaller biotech, we sought to better understand the landscape of PREA PMRs for antibiotics and identify strategies to improve PMR study completion.

## 2. Results

### 2.1. Overview of Pediatric Post-Marketing Requirements

Eighteen antibiotics with at least one indication of acute bacterial skin and structural infections (ABSSSI); hospital-acquired, community-, or ventilator-associated bacterial pneumonia (CABP, HABP, and VABP, respectively); complicated urinary tract infection (cUTI); or complicated intra-abdominal infection (cIAI) were approved between 2009 and 2024. Of the approved antibiotics, the majority were in the beta-lactam class (including cephalosporins, N = 9, 50%). There were three lipoglycopeptide antibiotics (16.67%), two tetracycline antibiotics (11.1%), and a single antibiotic that were approved in each of the following four classes: aminoglycoside, oxazolidinone, pleuromutilin, and quinolone. The 18 antibiotics had 53 associated PMRs required under PREA, with a median of 3 PMRs (range 1–5 PMRs) for each antibiotic. One PMR (1529-001, telavancin), originally required under PREA for its ABSSSI indication, was non-descriptive and excluded from further analysis [14].

Of the 52 remaining PMRs, 19 (36.5%) were related to the indication of ABSSSI, 12 (23.1%) to cUTI, 8 (15.4%) to CABP, 8 (15.4%) to HABP, 4 (7.7%) to cIAI, and 1 (1.9%) to *Staphylococcus aureus* bloodstream infections (SAB). The 52 PMRs specified 24 Phase 1 studies, 32 Phase 2 studies, and 2 studies that could not be categorized as either Phase 1 or Phase 2 studies. Some PMRs, such as 2809-1 for ceftolozane-tazobactam, specified two studies: a single-dose PK trial followed by a randomized controlled trial [15]. PMRs for sulbactam-durlobactam and cefepime-enmetazobactam did not have a dose-finding study specified. Ceftibiprole has been approved in Europe for the treatment of CABP and had a pediatric trial completed with labelling [16]. The FDA PMRs for ceftobiprole are Phase 2 studies in the new indications of SAB and ABSSSI [17,18,19]. Delafloxacin was the only antibiotic to have no PMRs under PREA for one indication (ABSSSI) and have PMRs under PREA for another indication (CABP) [20,21].

**Figure 1:**
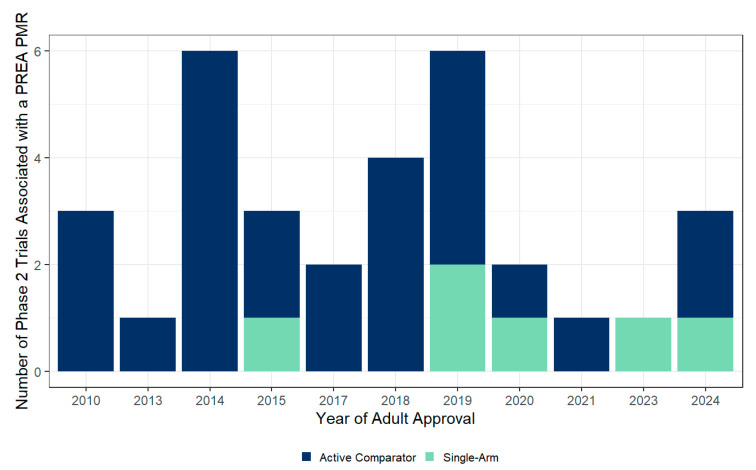
Trends in the utilization of single-arm study design for pediatric antibiotic trials under PREA. A summary of the number of Phase 2 studies associated with a PREA PMR that had an active comparator or single-arm design.

Before 2015, there were no single-arm, non-comparative studies with the primary endpoints of PK and safety that were accepted by the FDA, whereas, after 2015, one or two such studies have been accepted by the FDA per year (Figure 1). The full set of PMRs (including ongoing PMRs) are listed with cross-reference to clinicaltrials.gov and PubMed Identifiers (PMIDs) in Table S1 of the Supplementary Materials.

#### 2.1.1. Post-Marketing Requirements for Infants and Neonates

In total, there were 31 PMRs which specified the study of antibiotics in a dedicated neonatal and young infant subgroup (defined as children aged from birth to less than 3 months, from a regulatory perspective). A total of 15 antibiotics (all except eravacycline, omadacycline, and delafloxacin) that received PMRs under PREA were also mandated to be studied in neonates [22,23]. There were 33 studies corresponding to the 31 PMRs, as some PMRs described two studies (dose finding and Phase 2 in a single PMR). Of the 33 studies, 14 were Phase 1 dose-finding studies and 19 were Phase 2 trials. The PMRs for sulbactam-durlobactam and cefepime-enmetazobactam did not specify a single-dose PK (dose-finding) study prior to the evaluation of multiple doses in neonates.

#### 2.1.2. Waivers to Release Requirement of Antibiotic Investigation in Children

In the public domain, two antibiotics received a waiver or partial waiver to be released from the requirement to complete the initially agreed upon PMRs under PREA. The FDA agreed to release the sponsor from PMR 1692-004 (ceftaroline, neonatal cerebro-spinal fluid PK study) because the study was not considered feasible [24]. Telavancin was released from further study in children less than 6 years of age due to an unfavorable risk-benefit compared to other available antibiotics for ABSSSI in that patient population [25]. There may be more modifications of PMRs under PREA that are currently in negotiations or not yet disclosed to the public. Some antibiotics were released from the requirement to be studied in children at the time of approval. An example is delafloxacin, for which the FDA waived the requirement to study it for the indication of ABSSSI in all pediatric populations, as risk-benefit considerations did not support the use of delafloxacin for ABSSSI in children.

### 2.2. Pediatric Approvals

A total of 6 of 18 antibiotics (33.3%) achieved pediatric approval in at least one age group (ceftobiprole, ceftaroline, ceftolozane-tazobactam, dalbavancin, tedizolid, and cetazidime-avibactam). All six antibiotics were marketed by large pharmaceutical organizations, with a median time to pediatric approval of 5.8 years (range 0 to 7.34 years) from the adult approval date. Ceftolozane-tazobactam and dalbavancin achieved approval for patients aged from birth to less than 18 years at one time with a single supplemental NDA (sNDA) [26,27]. Other antibiotics, such as ceftaroline and cetazidime-avibactam, achieved pediatric approval for patients from birth to less than 18 years with a staged approach and multiple sNDAs. Small- to medium-sized companies have not yet been successful in achieving pediatric antibiotic approval for labelling updates.

### 2.3. PREA PMR Associated Study Completion

Whereas above, the content of 52 PMRs were described in an overview, for the purpose of study completion analyses, a total of 8 additional PMRs were excluded (six exclusions for projected study completion dates on or after 31 December 2024, and two exclusions because the study type was not categorizable as Phase 1 or Phase 2). Of the remaining 44 PMRs in the analysis set, the median pediatric study follow-up time from adult approval was 5.3 years (range from 0.94 to 11.5 years) with a study completion rate of 54.5% (N = 24). Small- and medium-sized companies had a study completion rate of 10% (N = 2/20) over a median of 6.44 years of follow-up time (range from 2.56 to 11.5 years). Large pharmaceutical corporations had a significantly higher study completion rate of 91.6% (N = 22/24; adjusted hazard ratio 20.3 95% CI, from 5.02 to 82.4) over a median follow-up time of 4.7 years (range 0.94 to 9.05 years). Small- to medium-sized pharmaceuticals did not complete a study including a neonatal population during the observation period, whereas large pharmaceutical organizations completed 85.7% (N = 12/14) of studies including a neonatal population. For large pharmaceutical organizations, studies including a neonatal population had longer completion times (median 5.62 years, range from 1.07 to 9.05 years, N = 12) compared to studies not including a neonatal population (median 3.75 years, range from 0.94 to 4.85 years, N = 10; Kruskal–Wallis test, df = 1, *p* = 0.006; adjusted hazard ratio 0.43 95% CI from 0.43 to 0.81). When categorizing by study type (Phase 1 vs. Phase 2), the median time to actual study completion was shorter for Phase 1 studies (3.26 years, range from 0.94 to 8.8 years, N = 7) than Phase 2 studies (4.85 years, range from 2.27 to 9.05 years, N = 15), which was not statistically significant (Kruskal–Wallis test, df = 1, *p* = 0.13; adjusted hazard ratio 0.54 95% CI from 0.26 to 1.09, *p*-value = 0.086).

Antibiotic indication, study phase, and the inclusion of neonates were not statistically significantly associated with study completion (Chi-square test, df = 4, 1 and 1, respectively; *p*-values 0.46, 0.85, and 0.3, respectively, N = 44). The association of antibiotic class and study completion was initially significant (Chi-squared test, df = 6, *p*-value = 0.013, N = 44) when combining large and smaller companies. However, this association was no longer significant when limiting to either all large pharmaceutical companies (Chi-squared test, df = 2, *p*-value = 0.64, N = 24) or all small/medium pharmaceutical companies (Chi-squared test, df = 5, *p*-value = 0.83, N = 20).

**Figure 2:**
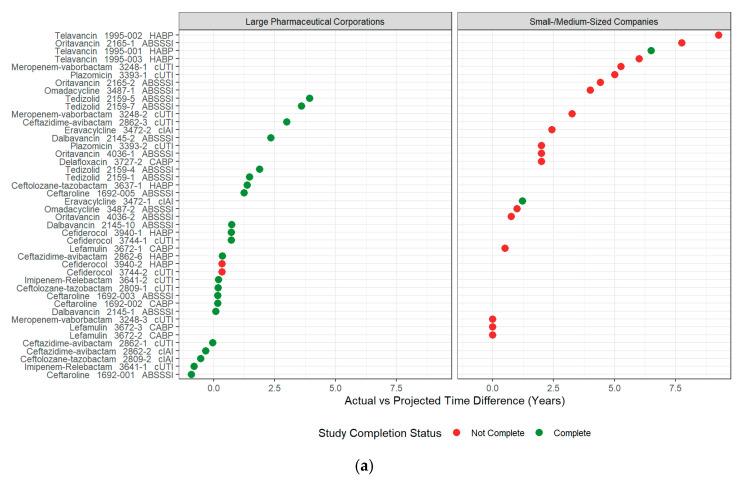

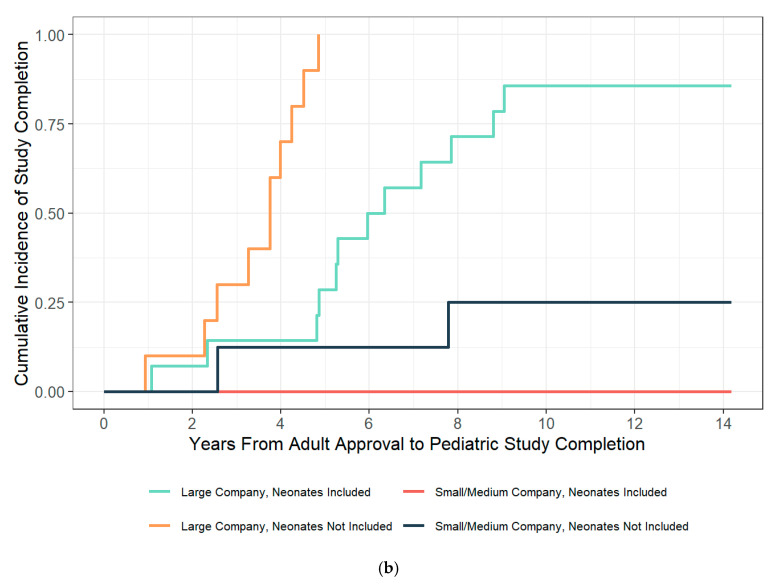
Completion status and time to complete a PMR-associated study. (**a**) Summary of PREA PMR-associated studies that have been completed, PREA PMR-associated studies that have not yet completed, and the difference between projected and actual time to complete a study associated with a PREA PMR. Circles represent the difference in actual vs. projected study completion time for studies associated with a PREA PMR (PMR number provided in y-label). Time differences that are less than 0 represent studies that were completed prior to the projected date; (**b**) Cumulative incidence of pediatric study completion from adult approval date by company size and inclusion of a neonatal population.

Antibiotic indication and antibiotic class were not significantly associated with time to completion (Kruskal–Wallis test, df = 2, 4, respectively; *p*-values = 0.89 and 0.31, respectively). Antibiotic indication and antibiotic class were not tested in multivariate Cox proportional hazard modeling due to small sample sizes, large effects associated with company size, and an uneven distribution of antibiotic indication and class amongst smaller and large pharmaceutical companies. Table S2 summarizes the multivariate model building process, and Table 1 summarizes the hazard ratios of the final multivariate model. Figure 2 summarizes individual PMR completion by company size versus the difference in proposed and actual completion times and the cumulative incidence of study completion over time categorized by company size and the inclusion of a neonatal study population.

## 3. Discussion

### 3.1. Discussion of Results

This study provides a comprehensive review of PMRs under PREA for antibiotics approved between 2009 and 2024. Large pharmaceutical organizations had a 91.6% completion rate for PMR associated studies and achieved pediatric approval for labelling updates for 75% of antibiotics (N = 6/8) in at least one pediatric age group over a median of 5.8 years (range from 0 to 7.3 years). In comparison, small- and medium-sized companies had a completion rate of 10% (N = 2/20) with no pediatric approvals over a similar time frame. With large pharmaceuticals completing the majority of PMRs, and with the shift of antibiotic development to smaller pharmaceutical companies, understanding the challenges smaller companies face and optimizing PMR completion plans are crucial.

The tough financial market for antibiotics is likely a large contributor to delays in PMR completion rates for small- to mid-sized pharmaceutical companies that depend on revenue from one or more antibiotic products. Total 5-year post-market costs after launch may range between $242 and 622 million, where the expected revenue from the approved antibiotic is generally lower by 10-fold [9], placing smaller companies at risk for insolvency [28]. Nevertheless, smaller companies, along with government agencies and global organizations, remain committed to pediatric drug development. Current funding opportunities from these agencies and organizations are a promising means to support continued antibiotic development and have the potential to enhance the completion rates of pediatric studies mandated by PREA for small- and medium-sized pharmaceutical companies [29].

Aside from funding, there are several considerations for study design that may enhance study completion rates for smaller companies. When developing antibiotics for pediatric populations, the FDA recommends that sponsors consider the extrapolation of efficacy from adequate and well-controlled clinical trials in adults. One approach to the extrapolation of efficacy is the PK and safety approach [30]. The PK and safety approach is acceptable for indications such as cIAI, cUTI, CABP, and ABSSSI [12], and it requires a PK study that identifies a pediatric dose to provide an exposure similar to that which was found to be effective in adults [30]. As such, PK and safety studies (including multiple-dose studies) may be designed as single-arm, non-comparative studies with smaller sample sizes based on precisely estimating PK parameters. Such studies with smaller enrollment and no treatment blinding requirements should be faster and less expensive to complete. Hwang et al. reported a 54.9% completion rate for studies with no comparator compared to 20.9% and 24.2% completion rates for studies with an active or placebo comparator, respectively [3]. As evidenced by Hwang et al., the utilization of the PK and safety extrapolation approach may help improve pediatric antibiotic study completion rates and time to completion.

Consistent with the PK and safety extrapolation approach, the FDA states, “a dedicated PK study is not always needed in every age group. For example, prior experience with dosing in adolescent patients has demonstrated that knowledge of adult dosing and appropriate dose scaling may be sufficient for some drugs with adequate justification” [13]. As such, sponsors should assess whether the clinical evaluation of an antibiotic is necessary in adolescents, or if the existing safety, PK, and efficacy data in a surrogate population of adults (e.g., low-weight adults) may serve as adequate evidence for approval in adolescents. Another approach is the inclusion of adolescents in the pivotal adult studies. Using this approach, gepotidacin was recently approved for use in treating uncomplicated UTIs in in female pediatric and adult patients aged 12 years of age and older [31].

A final consideration for improving study completion times is the use of adaptive integrative Phase 1/Phase 2 study designs. Traditionally pediatric drug development has proceeded sequentially (both by age group and by study type, i.e., Phase 1 completes prior to Phase 2). The World Health Organization (WHO), in collaboration with global regulators, pharmaceutical industry representatives, and the Global Antibiotic Research and Development Partnership (GARD-P), proposed that single-dose PK studies on background therapy in children offer limited value [32]. This collaborative group recommended the consideration of integrated Phase 1/Phase 2 study designs and potentially moving directly to a multiple-dose study based on tiered risk and prior knowledge of an antibiotic’s safety, PK, and efficacy profile in adults and children.

Our study found that, even for large pharmaceutical companies, studies including neonates did not complete in as timely of a fashion as studies not including neonates (adjusted hazard ratio 0.43 95% CI, from 0.23 to 0.81). This may be in part due to the sequential study of antibiotics in older children prior to evaluating the antibiotic in neonates. For lower risk antibiotics such as beta-lactams paired with novel beta-lactamase inhibitors, where the beta-lactam has been approved in young infants or neonates and has a history of extensive clinical use (i.e., cefepime, meropenem etc.), it may be reasonable to initiate adaptive Phase1/Phase2 studies in all pediatric age groups simultaneously. For antibiotics with a higher risk profile, it may be appropriate to initiate integrated adaptive Phase1/Phase2 studies gated by age group. Either approach has the potential to eliminate the delays awaiting completion of Phase 1, or evaluation in older age groups.

### 3.2. Limitations

Our analysis was limited by small samples size, and, therefore, the results should be interpreted with caution. Information such as the number of clinical trial sites and clinical trial dates was not always available and may be inaccurate in clinicaltrials.gov. To minimize errors in trial completion dates, we cross-referenced dates in clinicaltrials.gov to publications or FDA and sNDA reviews when available. We did not include the number of trial sites in this analysis. However, increasing the number of trial sites is associated with higher trial costs, so this may be accounted for in our analysis by differentiating between large and small/medium pharmaceutical companies.

Other factors for which our analysis could not account included the complexities of study enrollment timelines. Factors such as regulatory requirements of study approvals, site contracts and budgets, insurance, ensuring clinical supplies arrive to the sites, and appropriate site selection may all have an impact on enrollment timelines, independent of a planned study sample size [33].

Only certain information related to drug approvals and commitments are readily accessible by the public. Typically, the initial PMR and current PMR are available to the public. Sponsors may update study designs or request a deferral extension in consultation with the FDA as new evidence emerges or unforeseen challenges arise [34,35]. These updates are not always reflected as an updated PMR in the public domain, especially if the sponsor is currently in negotiations with the FDA. Therefore, our research may underestimate the use of single-arm, non-comparative trials or other innovative approaches.

A further limitation is the absence of a standard definition for large vs. small/medium pharmaceutical companies. Complex ownership structures and transaction histories can complicate these classifications. For example, Cipla USA acquired plazomicin from Achaogen in 2019, but Achaogen, a smaller company, owned the drug for much of the relevant period from adult approval to initial PMR study completion deadlines. Therefore, plazomicin was categorized as being owned by small/medium for this study. Such complexities highlight the need for potential reclassification in future research.

Finally, there was insufficient data to determine whether single-arm, non-comparator Phase 2 antibiotic studies complete faster than larger randomized studies with an active comparator. Only two single-arm Phase 2 studies were completed, both of which were studies including neonates, which generally take longer than studies without neonates.

## 4. Materials and Methods

### 4.1. Inclusion Criteria for Antibiotics and Search Method

Antibiotics were included if they were FDA-approved between 2009 and 2024 with at least one of the following indications: CABP, HABP, VABP, cUTI, cIAI, or ABSSSIs. FDA approval letters and FDA reviews of pediatric studies conducted under PREA were reviewed [36,37]. Specific FDA approval letters and FDA reviews of pediatric studies are cited in the text and tables. Where possible, pediatric studies conducted under PREA requirements were cross-referenced to trials registered at clinicaltrials.gov [38], summarized in Table S1 in the Supplementary Materials.

### 4.2. Data Extraction and Definitions

Information extracted from approval letters and clinicaltrials.gov included PMR number, adult approval date, proposed timeline for study completion from the adult approval date, actual timeline for study completion from the adult approval date, pediatric approval date, study phase, antibiotic indication, inclusion of neonatal population (defined as a dedicated subgroup of children aged from birth to <3 months), the use of an active comparator, and sponsor at the time of approval. The status of a final study report is often not disclosed in the public domain and often follows study completion requirement by 6 months or more. Therefore, the study completion date was used as a surrogate for PMR completion. Study completion was determined by cross-referencing a study to clinicaltrials.gov and extracting the actual completion date reported.

For the purpose of this research, a study was considered to be Phase 1 if its main goal was to inform further pediatric development studies. Phase 1 studies were usually single-dose PK studies. Phase 2 studies were defined as studies that generated the data required by the FDA for sNDA. Phase 2 pediatric antibiotic studies may be single-arm non-comparative trials with the primary endpoints of PK and safety, or randomized controlled trials more similar to traditional Phase 2 studies in adults. When a PMR included two studies, such as PMRs 2809-1 for ceftolozane-tazobactam (randomized control trial, with dose to be informed by a single-dose PK study), the categorizations and dates were based on the study that would ultimately fulfill the PMR [15]. For example, PMR 2809-1 for ceftolozane-tazobactam was categorized as Phase 2 for the purpose of PMR completion and time to completion; however, for study counting purposes, the Phase 1 study was included in the overall count.

Companies were categorized as large pharmaceutical organizations if the company’s total revenue was greater than or equal to $1 billion within one year of the initial adult antibiotic approval. The transaction history of antibiotics is complex and beyond the scope of this research to describe. The full categorization of companies is presented in Table S2 in the Supplementary Materials.

### 4.3. Statistical Analyses

All analyses were performed using R v4.4.0 with RStudio 2024.04.2 Build 764 (Posit Software, PBC, Boston, MA, USA) utilizing the tidyverse, tidycmprsk, and survival packages [39,40,41]. Descriptive analyses, including Chi-square and Kruskal–Wallis tests, were conducted to explore univariate associations between variables. Multivariate Cox proportional hazards regression was then employed for the primary statistical inference to assess the independent effects of covariates on the outcome of study completion. A forward stepwise selection approach was used to build the multivariate Cox proportional hazards model. Plausible covariates were considered for inclusion based on initial descriptive analyses. At each step, the log-likelihood ratio test was used to compare nested models, retaining covariates if they significantly improved model fit (*p* < 0.05, corresponding to a change of approximately 3.84 in the −2 log-likelihood). Studies were followed from the adult approval date until completion or through 31 December 2024, whichever happened first (allowing for at least 3 years of follow-up time). Studies not completed by 31 December 2024, were censored at the end of follow-up. A *p*-value of < 0.05 was considered the threshold for statistical significance.

Studies with a projected completion date on or after 31 December 2024 were excluded from the analysis due to limited follow-up time (PMRs 3762-2 and 3762-3 for lefamulin, PMR 4452-1 for sulbactam-durlobactam, PMR 4582-1 for cefepime-enmetazobactam, and PMRs 4612-1 and 4612-2 for ceftobiprole were excluded for this reason). PMR (1529-001, telavancin), originally required under PREA for the ABSSSI indication, was non-descriptive and excluded from further analysis. PMR 1692-004 for ceftaroline was a CNS PK study in neonates that was terminated early in consultation with the FDA due to the impracticability of the study. PMR 3721-1 for delafloxacin was a bioavailability study in adults to test a taste-masked pediatric formulation and were excluded from the completion rate and projected and completion time analyses.

## 5. Conclusions

Compared to larger organizations, smaller pharmaceutical companies have experienced difficulty in PREA PMR antibiotic study completion, which may be related to financial difficulties in the challenging market for antibiotics. To improve PMR study completion, smaller companies require continued financial support and innovation in study design. For pediatric antibiotic development, the FDA accepts the extrapolation of efficacy from well-conducted randomized adult trials (i.e., PK and safety approach). Therefore, sponsors should consider the use of single-arm, non-comparative PK and safety studies to reduce the size and scope of trials. Sponsors should also assess whether the evaluation of an antibiotic is necessary in adolescents, or if data in a surrogate population of adults (e.g., low-weight adults) may serve as adequate evidence for adolescent approval. When appropriate, sponsors should consider using single-arm, non-comparative study designs and streamlined Phase 1/Phase 2 study approaches.

## Figures and Tables

**Table 1:** Summary of final multivariate Cox proportional hazards model.

Covariate	Hazard Ratio ^1^	95% Confidence Interval	*p*-Value
Company Size	20.3	5.02 to 82.4	<0.001
Inclusion of Neonatal Population in Study	0.43	0.23 to 0.81	0.009

^1^ Hazard ratios less than 1 indicate increased risk of delayed study completion; hazard ratios greater than 1 indicate reduced risk.

## Data Availability

Data may be made available on a case-by-case basis upon reasonable request.

## Supplementary Materials

The following supporting information can be downloaded at: https://www.mdpi.com/article/10.3390/antibiotics14060583/s1, Table S1: Landscape of Post-Marketing Requirements Under the Pediatric-Research Equity Act for Antibiotics Approved from 2009 to 2024. Table S2: Model Selection Summary for Cox Proportional Hazards Analysis. Table S3: Financial Performance of Market Authorization Holders of FDA-Approved Antibiotics. The following references were cited in the Supplementary Materials: [14,15,16,17,20,21,22,23,25,42,43,44,45,46,47,48,49,50,51,52,53,54,55,56,57,58,59,60,61,62,63,64,65,66,67,68,69,70,71,72,73,74,75,76,77,78,79,80,81,82,83,84,85,86,87,88,89,90,91,92,93,94,95,96,97,98,99,100,101,102,103,104,105,106,107,108,109,110,111,112,113,114,115,116,117,118,119,120,121,122,123,124,125,126,127,128,129,130,131,132,133,134,135,136,137,138,139,140,141,142,143,144,145,146,147,148,149,150,151,152].

## Abbreviations

The following abbreviations are used in this manuscript:

ABSSSI	Acute Bacterial Skin and Structural Infections
CABP	Community Associated Bacterial Pneumonia
cIAI	Complicated Intra-abdominal Infections
cUTI	Complicated Urinary Tract Infections
FDA	Food and Drug Administration
GARDP	Global Antibiotic Research and Development Partnership
HABP	Hospital Associated Bacterial Pneumonia
ICH	International Council for Harmonisation
NDA	New Drug Application
PMID	PubMed Identifier
PMR	Post-marketing requirement
PREA	Pediatric Research Equity Act
sNDA	Supplemental New Drug Application
VABP	Ventilator Associated Bacterial Pneumonia
WHO	World Health Organization
