# Supplementary material for: Landscape of Post-Marketing Requirements Under the Pediatric Research Equity Act for Antibiotics from 2009–2024
Source: Antibiotics (Basel). 2025 Jun 6;14(6):583. doi: 10.3390/antibiotics14060583 (PMC12189388; doi:10.3390/antibiotics14060583)
Supplement: Supplementary file 1 [file antibiotics-14-00583-s001.zip › antibiotics-3645630-supplementary.pdf]

Table S1: Landscape of Post-Marketing Requirements Under the Pediatric-Research Equity Act for Antibiotics Approved from 2009 to 2024

Antibiotic	Adult Approval Year/Indication(s)	PREA /BPCA Requirements	Proposed timeline for study completion	Actual Timeline	Pediatric Approval Date(s)	Regulatory References	ClinicalTrials.gov References	Peer Review Publication References
Telavancin	2009: cSSSI<br>2013: HABP/VABP	1529-001: Not specified < 18 years<br>1995-001: SD PK 1 to < 18 years<br>1995-002: SD PK 0 to 1 years<br>1995-003: RCT birth to < 18 years	1529-001: 12/2014[1]<br>1995-001: 09/2014<br>1995-002: 09/2015<br>1995-003: 12/2018	1529-001: No Info<br>1995-001: 03/2021<br>1995-002: Waived[2]<br>1995-003: Not Started		Adult Approval cSSSI [1]<br>Adult Approval HABP/VABP [2]	1995-001: NCT02013141 [3]	1995-001: PMID: 37815398 [4]
Ceftaroline	2010: ABSSSI, CABP	1692-001: SD PK < 12 years<br>1692-002: RCT, CABP < 18 years<br>1692-003: RCT, ABSSSI < 18 years<br>1692-004: CSF PK, < 2 months of age<br>1692-005: RCT, CABP/ABSSSI < 2 months of age	1692-001: 01/2014<br>1692-002: 05/2014<br>1692-003: 05/2014<br>1692-004: 03/2016<br>1692-005: 09/2016	1692-001: 02/2013<br>1692-002: 07/2014<br>1692-003: 07/2014<br>1692-004: 01/2018[3]<br>1692-005: 12/2017[3]	05/2016: ABSSI, CABP, 2 months to < 18 years<br>09/2019: ABSSI, CABP, < 2 months	Adult Approval [5]<br>Pediatric Approval (> 2 months) [6]<br>Pediatric Approval (< 2 months) [7]	1692-001: NCT01298843 [8]<br>1692-002: NCT01530763 [9]<br>1692-003: NCT01400867 [10]<br>1962-005: NCT02424734 [11]	1692-002: PMID: 27078119 [12]<br>1692-003: PMID: 27164462 [13]<br>1962-005: PMID: 32091493 [14]
Ceftolozane-Tazobactam	2014: cIAI, cUTI<br>2019: HABP/VABP	2809-1: RCT, cUTI, < 18 years<br>2809-2: RCT, cIAI, < 18 years<br>3637-1: PK, birth to < 18, HABP/VABP	2809-1: 09/2020<br>2809-2: 09/2020<br>3637-1: 04/2023	2809-1: 12/2020[4]<br>2809-2: 03/2020[4]<br>3677-1: 09/2024	04/2022: cIAI, cUTI, birth to < 18 years	Adult Approval cIAI, cUTI [15]<br>Adult Approval HABP/VABP [16]<br>Pediatric Approval cIAI, cUTI [17]	2809-1: NCT03230838 [18]<br>2809-2: NCT03217136 [19]<br>3677-1: NCT04223752 [20]	2809-1: PMID: 36689671 [21]<br>2809-2: PMID: 37000942 [22]
Dalbavancin	2014: ABSSSI	2145-1: SD PK 3 months to < 12 years<br>2145-2: SD PK birth to 3 months<br>2145-10[2]: RCT birth to < 18 years	2145-1: 03/2015<br>2145-2: 11/2016<br>2145-10: 12/2019	2145-1: 03/2017<br>2145-2: 04/2019<br>2145-10: 09/2020[5,6]	07/2021: ABSSSI, birth to < 18 years	Adult Approval [23]<br>Pediatric Approval [24]	2145-1: NCT01946568 [25]<br>2145-2: NCT02688790 [26]<br>2145-10: NCT02814916 [27]	2145-10: PMID: 36476623 [28]
Oritavancin	2014: ABSSSI<br>2021: New formulation	2165-1: SD PK birth to < 18 years<br>2165-2: RCT birth to < 18 years<br>4036-1: SD PK birth to < 18 years (new formulation)<br>4036-2: RCT birth to < 18 years (new formulation)	2165-1: 03/2017<br>2165-2: 07/2020<br>4036-1: 12/2022<br>4036-2:03/2024	2165-1: Recruiting<br>2165-2: Recruiting<br>4036-1: Recruiting<br>4036-2: Recruiting		Adult Approval Orbactiv [29]<br>Adult Approval Kimyrsa [30]	2165-1: NCT02134301 [31]<br>2165-2: NCT05599295 [32]<br>4036-1: NCT02134301 [31]<br>4036-2: NCT05599295 [32]	
Tedizolid	2014: ABSSSI	2159-1: RCT 12 to < 18 years IV to PO<br>2159-4: SD PK 2 to 12 years<br>2159-5: SD PK < 2 years<br>2159-7[3]: RCT birth to 12 years IV to PO	2159-1: 03/2017<br>2159-4: 01/2017<br>2159-5: 04/2019<br>2159-7: 08/2021[7]	2159-1: 09/2018<br>2159-4: 12/2018<br>2159-5: 04/2023<br>2159-7: 07/2023	06/2020: ABSSSI, 12 to < 18 years	Adult Approval [33]<br>Pediatric Approval 12 to < 18 [34]<br>Consolidation of PMRs [35]	2159-1: NCT02276482 [36]<br>2159-4: NCT02750761 [37]<br>2159-5: NCT03217565 [38]<br>2159-7: NCT03176134 [39]	2159-1: PMID: 33395210 [40]<br>2159-1: PMID: 33710976 [41]
Ceftazidime-Avibactam	2015: cIAI, cUTI<br>2018: HABP/VABP	2862-1: RCT cUTI 3 months to < 18 years<br>2862-2: RCT cIAI 3 months to < 18 years<br>2862-3: SD PK birth to 3 months<br>2862-6: OL, SD PK 3 months to < 18 years H/VABP	2862-1: 09/ 2017<br>2862-2: 09/2017<br>2862-3: 12/2019<br>2862-6: 12/2020	2862-1: 09/2017<br>2862-2: 06/2017[4]<br>2862-3: 12/2022[4]<br>2862-6: 04/2021	03/2019: cIAI, cUTI, 3 months to < 18 years<br>12/2022: H/VABP, 3 months to < 18 years<br>01/2024: cIAI, cUTI, H/VABP, < 3 months	Adult Approval cIAI, cUTI [42]<br>Adult Approval HABP/VABP [43]<br>Pediatric Approval cIAI, cUTI [44]<br>Pediatric Approval HABP/VABP [45]<br>Pediatric Approval < 3 months [46]	2862-1: NCT02497781[47]<br>2862-2: NCT02475733 [48]<br>2862-3: NCT04126031 [49]<br>2862-6: NCT04040621 [50]	2862-1: PMID: 31335570 [51]<br>2862-2: PMID: 31306396 [52]
Delafloxacin	2017: ABSSSI<br>2019: CABP	ABSSSI requirements waived<br>3727-1: BA study for oral formulation peds<br>3727-2: SD PK 2 months to < 18 years<br>3727-3: OL, RCT 2 months to < 18 years	3727-1: 04/2020<br>3727-2: 12/2022<br>3727-3: 02/2026	3727-1: 09/2024<br>3727-2: Not Started<br>3727-3: Not Started		Adult Approval ABSSSI [53]<br>Adult Approval CABP [54]	3727-1: NCT06612255 [55]	
Meropenem-Vaborbactam	2017: cUTI	3248-1: SD PK birth to < 18<br>3248-2: RCT 3 months to 18 years<br>3248-3: OL, AC, birth to 3 months	3248-1: 09/2019<br>3248-2: 09/2021<br>3248-3: 12/2024	3248-1: Recruiting<br>3248-2: Recruiting<br>3248-3: Not Started		Adult Approval [56]	3248-1: NCT02687906 [57]<br>3248-2: NCT06672978 [58]	
Eravacycline	2018: cIAI	3472-1: SD PK 8 to < 18 years<br>3472-2: RCT 8 to < 18 years	3472-1: 12/2019<br>3472-2: 01/2023	3472-1: 03/2021<br>3472-2: Recruiting		Adult Approval [59]	3472-1: NCT03696550 [60]<br>3472-2: NCT06794541 [61]	
Omadacycline	2018: CABP, ABSSSI	3487-1: SD PK 8 to 17 years<br>3487-2: RCT 8 to 17 years ABSSSI<br>3487-3: RCT 8 to 17 years CABP	3487-1: 12/2020<br>3487-2: 12/2023<br>3487-3: 12/2025	3487-1: Recruiting<br>3487-2: Not Started<br>3487-3: Not Started		Adult Approval [62]	3487-1: NCT05217537 [63]	
Plazomicin	2018: cUTI	3393-1: MD PK birth to < 18 years<br>3393-2: RCT PK/Safety birth to < 18 years	3393-1: 12/2019<br>3393-2: 12/2022	3393-1: Not Started<br>3393-2: Not Started		Adult Approval [64]		
Cefiderocol	2019: cUTI<br>2020: HABP/VABP	3744-1: RCT, 3 months to 18 years<br>3744-2: OL, SA, birth to 3 months<br>3940-1: OL, RCT, 3 months to < 18 years<br>3940-2: OL, SA, birth to 3 months	3744-1: 12/2023<br>3744-2: 08/2024<br>3940-1: 12/2023<br>3940-2: 08/2024	3744-1: 09/2024[4]<br>3744-2: Recruiting[4]<br>3940-1: 09/2024[4]<br>3940-2: Recruiting[4]		Adult Approval cUTI [65]<br>Adult Approval H/VABP [66]	3744-1: NCT04215991 [67]<br>3744-2: NCT06086626 [68]<br>3940-1: NCT04215991 [67]<br>3940-2: NCT06086626 [68]	
Imipenem-Relebactam	2019: cUTI, cIAI	3641-1: SD PK birth to < 18 years<br>3641-2: OL, RCT, birth to < 18 years	3641-1: 05/2021<br>3641-2: 02/2024	3641-1: 08/2020<br>3641-2: 05/2024		Adult Approval [69]	3641-1: NCT03230916 [70]<br>3641-2: NCT03969901 [71]	3641-1: PMID: 37562063 [72]
Lefamulin	2019: CABP	3672-1: SD PK birth to < 18 years, IV<br>3672-2: SD PK birth to < 18, oral<br>3672-3: RCT, 2 months to < 18 years	3672-1: 06/2024<br>3672-2: 12/2024<br>3672-3: 12/2024	3672-1: Not Started<br>3672-2: Not Started<br>3672-3: Not Started		Adult Approval [73]		
Sulbactam – durlobactam	2023: HABP/VABP	4452-1: MD PK, birth to < 18 years	4452-1: 05/2028	4452-1: Recruiting		Adult Approval [74]	4452-1: NCT06801223 [75]	
Cefepime - enmetazobactam	2024: cUTI	4582-1: OL, SA, PK birth to < 18 years	4582-1: 02/2026	4582-1: Recruiting		Adult Approval [76]	4582-1: NCT05826990 [77]	
Ceftobiprole	2024: ABSSSI, SAB, CABP	4612-1: RCT, birth to < 18 years, ABSSSI<br>4612-2: RCT, birth to < 18 years, SAB	4612-1: 10/2027<br>4612-2: 10/2028	4612-1: Not Started<br>4612-2: Not Started	04/2024, CABP, 3 months to < 18 years	Adult Approval [78]		

1. PMR 1529-001 was non-descript, and only the final report date was available in the approval letter.
2. Per PMID: 37815398, Telavancin received a partial waiver to release the requirement to study the antibiotic in children < 6 years of age.
3. Agency released sponsor from 1692-004 and recommended sNDA submission with popPK report with partial results from requirement 1692-005
4. These PMRs specified that the dosing for the phase 2 portion would be informed from a single-dose PK study, however the single-dose PK study was not a separate PMR.
5. Consolidation of original dalbavancin PMR's 2145-3 and 2145-4, study completion date is based on the original proposed date for PMR 2145-4
6. Dalbavancin had discrepant trial end date, was in 2024 per trials.gov but FDA review reported study completion in 2020
7. Consolidation of original tedizolid PMR's 2159-2 and 2159-3

Abbreviations: AC = active comparator, OL = open-label, MD = multiple dose, PK = pharmacokinetic, PMR = Post-marketing requirement, RCT = randomized controlled trial, SD = single dose

Table S2: Model Selection Summary for Cox Proportional Hazards Analysis

Model Number	Model Description	-2LL	Δ-2LL/Compared to	p-value (LLR Test)	Decision
1	Base	146.2			-
2	Base + Company size	117.7	28.5/Model 1	<0.0001	Accept
**3**	**Model 2 + Neonate**	**113.8**	**3.93/Model 2**	**0.047**	**Accept (Final Model)[1]**
4	Model 2 + Study Phase	115.47	2.23/Model 2	0.14	Reject[2]
5	Model 3 + Study Phase	112.09	1.7/Model 3	0.2	Reject

[1]The Schoenfeld residuals test yielded p-values of 0.98 (Company Size), 0.69 (Neonate), and 0.92 (global), indicating no significant violations of the proportional hazards assumption.

[2]Although Model 4 did not meet the selection criteria based on the likelihood ratio test (LLR), the hazard ratio for study phase was 0.49 (95% CI: 0.25–0.98, p = 0.043), suggesting Phase 2 studies may take longer to complete than Phase 1 studies. Given the plausibility of this association, study phase was retained in Model 5. However, Model 5 was also rejected based on the LLR test, and study phase was no longer statistically significant (hazard ratio 0.54, 95% CI: 0.26–1.09, p = 0.21).

Abbreviations: 2LL = 2 log-likelihood, LLR =log-likelihood ratio

Table S3: Financial Performance of Market Authorization Holders of FDA-Approved Antibiotics

Initial Adult Approval Year	Product	Sponsor at NDA[1]	Sponsor at NDA Total Revenue within 1 year of NDA (US Dollars in Millions)	Current Product Distributor[1]	Current Product Distributor Total Revenue Fiscal Year 2024 (US Dollars in Millions)	Company Size	References
2009	Telavancin	Theravance	24.4	Cumberland	38	Small/Medium	[1,79–81]
2010	Ceftaroline	Cerexa	3920	Allergan	16,544	Large	[5,82,83]
2014	Ceftolozane -Tazobactam	Cubist	1100	Merck	64,200	Large	[15,84–86]
2014	Dalbavancin	Durata	8700	Abbvie	56,330	Large	[23,87–90]
2014	Oritavancin	The Medicines Company	687.9	Melinta	Not available	Small/Medium	[29,91,92]
2014	Tedizolid	Cubist	1100	Merck	64,200	Large	[33,85,86,93]
2015	Ceftazidime - avibactam	Forest	3100	Abbvie	56,330	Large	[42,90,94,95]
2017	Delafloxacin	Melinta	34	Melinta	34	Small/Medium	[96–98]
2017	Meropenem - vaborbactam	Rempex	34	Melinta	34	Small/Medium	[56,98,99]
2018	Eravacycline	Tetraphase	18.9	Tetraphase	Not Available	Small/Medium	[59,100,101]
2018	Omadacycline	Paratek	12.61	Paratek	Not Available	Small/Medium	[62,102,103]
2018	Plazomicin	Achaogen	11.2	Cipla USA	3180	Small/Medium[2]	[64,104–106]
2019	Cefiderocol	Shionogi	3140	Shionogi	2960	Large	[65,107,108]
2019	Imipenem - Relebactam	Merck	42,300	Merck	64,200	Large	[69,86,109,110]
2019	Lefamulin	Nabriva	6.5	Nabriva	Not Available	Large	[73,111,112]
2023	Sulbactam - durlobactam	Entasis[3]	310.5	La Jolla[3]	358.71	Small/Medium	[74,113–115]
2024	Cefepime - enmetazobactam	Allecra	No Revenue	Allecra	Not Available	Small/Medium	[76,116–118]
2024	Ceftobiprole	Basilea	183.02	Basilea	242.15	Small/Medium	[78,119,120]

1. Many antibiotic products and companies listed in this table have a complex transaction history, which is beyond the scope of this research to describe. Given the complex transaction histories, categorization of company size may not be static. Sponsor at NDA and current product distributor were extracted from the approval letter and the most recent product label available at drugs@FDA. Specific references are cited in the table.
2. Cipla USA Inc., a subsidiary of Cipla acquired rights to plazomicin in 2019. The financial support from Cipla to its subsidiary Cipla USA is unclear and plazomicin was owned by Achaogen for a significant portion of time from approval until the initial proposed PREA PMR deadlines. Therefore, plazomicin was classified to be owned by a small/medium pharmaceutical company.
3. Innoviva acquired Entasis and La Jolla in 2022.

References

1. Food and Drug Administration Telavancin New Drug Application Approval Letter 2009.
2. Food and Drug Administration Telavancin Supplemental New Drug Application Approval Letter 2013.
3. Cumberland Pharmaceuticals Telavancin Pediatric PK Study (Ages >12 Months to 17 Years) Available online: https://clinicaltrials.gov/study/NCT02013141?cond=NCT02013141&rank=1 (accessed on 23 July 2024).
4. Bradley, J.S.; Goldman, J.L.; James, L.P.; Kaelin, B.; Gibson, B.H.Y.; Arrieta, A. Pharmacokinetics and Safety of a Single Dose of Telavancin in Pediatric Subjects 2-17 Years of Age. *Antimicrob Agents Chemother* **2023**, *67*, e0098723, doi:10.1128/aac.00987-23.
5. Food and Drug Administration Ceftaroline New Drug Application Approval Letter 2010.
6. Food and Drug Administration Ceftaroline Supplemental New Drug Application 2 Months to Less Than 18 Years of Age Approval Letter 2016.
7. Food and Drug Administration Ceftaroline Supplemental New Drug Application Birth to Less Than 2 Months of Age Approval Letter 2019.
8. Forest Laboratories Study of Blood Levels of Ceftaroline Fosamil in Children Who Are Receiving Antibiotic Therapy in the Hospital Available online: https://clinicaltrials.gov/study/NCT01298843?cond=NCT01298843&rank=1 (accessed on 23 July 2024).
9. Forest Laboratories A Multicenter, Randomized, Observer-Blinded, Active-Controlled Study Evaluating the Safety, Tolerability, Pharmacokinetics, and Efficacy of Ceftaroline Versus Ceftriaxone in Pediatric Subjects With Community-Acquired Bacterial Pneumonia Requiring Hospitalization Available online: https://clinicaltrials.gov/study/NCT01530763 (accessed on 20 February 2025).
10. Forest Laboratories Safety and Efficacy Study of Ceftaroline Versus a Comparator in Pediatric Subjects With Complicated Skin Infections Available online: https://clinicaltrials.gov/study/NCT01400867?cond=NCT01400867&rank=1 (accessed on 23 July 2024).
11. Pfizer Safety, Tolerability and Efficacy of Ceftaroline in Paediatrics With Late-Onset Sepsis Available online: https://clinicaltrials.gov/study/NCT02424734?cond=NCT02424734&rank=1#study-overview (accessed on 23 July 2024).
12. Cannavino, C.R.; Nemeth, A.; Korczowski, B.; Bradley, J.S.; O'Neal, T.; Jandourek, A.; Friedland, H.D.; Kaplan, S.L. A Randomized, Prospective Study of Pediatric Patients With Community-Acquired Pneumonia Treated With Ceftaroline Versus Ceftriaxone. *Pediatr Infect Dis J* **2016**, *35*, 752–759, doi:10.1097/INF.0000000000001159.
13. Korczowski, B.; Antadze, T.; Giorgobiani, M.; Stryjewski, M.E.; Jandourek, A.; Smith, A.; O'Neal, T.; Bradley, J.S. A Multicenter, Randomized, Observer-Blinded, Active-Controlled Study to Evaluate the Safety and Efficacy of Ceftaroline Versus Comparator in Pediatric Patients With Acute Bacterial Skin and Skin Structure Infection. *Pediatr Infect Dis J* **2016**, *35*, e239-247, doi:10.1097/INF.0000000000001191.
14. Bradley, J.S.; Stone, G.G.; Chan, P.L.S.; Raber, S.R.; Riccobene, T.; Mas Casullo, V.; Yan, J.L.; Hendrick, V.M.; Hammond, J.; Leister-Tebbe, H.K. Phase 2 Study of the Safety, Pharmacokinetics and Efficacy of Ceftaroline Fosamil in Neonates and Very Young Infants With Late-Onset Sepsis. *Pediatr Infect Dis J* **2020**, *39*, 411–418, doi:10.1097/INF.0000000000002607.
15. Food and Drug Administration Ceftolozane - Tazobactam New Drug Application Approval Letter 2014.
16. Food and Drug Administration Ceftolozane - Tazobactam Supplemental New Drug Application Approval Letter Available online: https://www.accessdata.fda.gov/drugsatfda_docs/appletter/2019/206829Orig1s008ltr.pdf (accessed on 16 July 2024).
17. Food and Drug Administration Ceftolozane - Tazobactam Supplemental New Drug Application Birth to Less than 18 Years of Age Approval Letter 2022.
18. Merck Sharp & Dohme LLC MK-7625A Versus Meropenem in Pediatric Participants With Complicated Urinary Tract Infection (cUTI) (MK-7625A-034) Available online: https://clinicaltrials.gov/study/NCT03230838?cond=NCT03230838&rank=1 (accessed on 23 July 2024).
19. Merck Sharp & Dohme LLC MK-7625A Plus Metronidazole Versus Meropenem in Pediatric Participants With Complicated Intra-Abdominal Infection (cIAI) (MK-7625A-035) Available online: https://clinicaltrials.gov/study/NCT03217136?cond=NCT03217136&rank=1 (accessed on 23 July 2024).
20. Merck Sharp & Dohme LLC Safety and Pharmacokinetics of Ceftolozane/Tazobactam in Pediatric Participants With Nosocomial Pneumonia (MK-7625A-036) Available online: https://clinicaltrials.gov/study/NCT04223752?cond=NCT04223752&rank=1 (accessed on 23 July 2024).
21. Roilides, E.; Ashouri, N.; Bradley, J.S.; Johnson, M.G.; Lonchar, J.; Su, F.-H.; Huntington, J.A.; Popejoy, M.W.; Bensaci, M.; De Anda, C.; et al. Safety and Efficacy of Ceftolozane/Tazobactam Versus Meropenem in Neonates and Children With Complicated Urinary Tract Infection, Including Pyelonephritis: A Phase 2, Randomized Clinical Trial. *Pediatr Infect Dis J* **2023**, *42*, 292–298, doi:10.1097/INF.0000000000003832.
22. Jackson, C.-C.A.; Newland, J.; Dementieva, N.; Lonchar, J.; Su, F.-H.; Huntington, J.A.; Bensaci, M.; Popejoy, M.W.; Johnson, M.G.; De Anda, C.; et al. Safety and Efficacy of Ceftolozane/Tazobactam Plus Metronidazole Versus Meropenem From a Phase 2, Randomized Clinical Trial in Pediatric Participants With Complicated Intra-Abdominal Infection. *Pediatr Infect Dis J* **2023**, *42*, 557–563, doi:10.1097/INF.0000000000003911.
23. Food and Drug Administration Dalbavancin New Drug Application Approval Letter 2014.
24. Food and Drug Administration Dalbavancin Supplemental New Drug Application Birth to Less Than 18 Years of Age Approval Letter 2021.
25. Durata Therapeutics Inc., an affiliate of Allergan plc A Single Dose Study To Investigate The Pharmacokinetics and Safety Of Dalbavancin In Hospitalized Children Aged 3 Months to 11 Years Available online: https://clinicaltrials.gov/study/NCT01946568?cond=NCT01946568&rank=1 (accessed on 23 July 2024).
26. AbbVie Study Evaluate the PK Profile of Dalbavancin in Infants and Neonates Patients With Known or Suspected Bacterial Infection Available online: https://clinicaltrials.gov/study/NCT02688790?cond=NCT02688790&rank=1 (accessed on 23 July 2024).
27. AbbVie Dalbavancin for the Treatment of Acute Bacterial Skin and Skin Structure Infections in Children, Known or Suspected to Be Caused by Susceptible Gram-Positive Organisms, Including MRSA Available online: https://clinicaltrials.gov/study/NCT02814916?cond=NCT02814916&rank=1 (accessed on 23 July 2024).

28. Giorgobiani, M.; Burroughs, M.H.; Antadze, T.; Carrothers, T.J.; Riccobene, T.A.; Patel, R.; Lin, T.; Stefanova, P. The Safety and Efficacy of Dalbavancin and Active Comparator in Pediatric Patients With Acute Bacterial Skin and Skin Structure Infections. *Pediatr Infect Dis J* **2023**, *42*, 199–205, doi:10.1097/INF.0000000000003798.
29. Food and Drug Administration Orbactiv New Drug Application Approval Letter 2014.
30. Food and Drug Administration Kimyrsa New Drug Application Approval Letter 2021.
31. Melinta Therapeutics, LLC Open-Label, Dose-Finding, Pharmacokinetics, Safety and Tolerability Study of Oritavancin in Pediatric Patients With Suspected or Confirmed Bacterial Infections Available online: https://clinicaltrials.gov/study/NCT02134301?cond=NCT02134301&rank=1 (accessed on 23 July 2024).
32. Melinta Therapeutics, LLC Study to Evaluate the Safety and Tolerability of Single-Dose Intravenous (IV) Oritavancin Available online: https://clinicaltrials.gov/study/NCT05599295?cond=NCT05599295&rank=1 (accessed on 23 July 2024).
33. Food and Drug Administration Tedizolid New Drug Application Approval Letter 2014.
34. Food and Drug Administration Tedizolid Supplemental New Drug Application 12 Years to Less Than 18 Years of Age Approval Letter 2020.
35. Merck Sharp & Dohme LLC US Postmarketing Requirements Status as of 12-Apr-2024 Available online: https://www.merck.com/wp-content/uploads/sites/124/2024/04/US-Postmarketing_Requirements_12-Apr-2024-FINAL.pdf (accessed on 25 February 2025).
36. Cubist Pharmaceuticals LLC, a subsidiary of Merck & Co., Inc. Study of Tedizolid Phosphate in Adolescents With Complicated Skin and Soft Tissue Infection (cSSTI) (MK-1986-012) Available online: https://clinicaltrials.gov/study/NCT02276482?cond=NCT02276482&rank=1 (accessed on 23 July 2024).
37. Merck Sharp & Dohme LLC A Study of Oral and Intravenous (IV) Tedizolid Phosphate in Hospitalized Participants, Ages 2 to <12 Years, With Confirmed or Suspected Bacterial Infection (MK-1986-013) Available online: https://clinicaltrials.gov/study/NCT02750761?cond=NCT02750761&rank=1 (accessed on 23 July 2024).
38. Merck Sharp & Dohme LLC A Pharmacokinetic Study of Tedizolid Phosphate in Pediatric Participants With Gram-Positive Infections (MK-1986-014) Available online: https://clinicaltrials.gov/study/NCT03217565?cond=NCT03217565&rank=1 (accessed on 23 July 2024).
39. Merck Sharp & Dohme LLC A Study of Safety and Efficacy of MK-1986 (Tedizolid Phosphate) and Comparator in Participants From Birth to Less Than 12 Years of Age With Acute Bacterial Skin and Skin Structure Infections (MK-1986-018) Available online: https://clinicaltrials.gov/study/NCT03176134?cond=NCT03176134&rank=1 (accessed on 23 July 2024).
40. Bradley, J.S.; Antadze, T.; Ninov, B.; Tayob, M.S.; Broyde, N.; Butterton, J.R.; Chou, M.Z.; De Anda, C.S.; Kim, J.Y.; Sears, P.S. Safety and Efficacy of Oral and/or Intravenous Tedizolid Phosphate From a Randomized Phase 3 Trial in Adolescents With Acute Bacterial Skin and Skin Structure Infections. *Pediatr Infect Dis J* **2021**, *40*, 238–244, doi:10.1097/INF.0000000000003010.
41. Arrieta, A.C.; Ang, J.Y.; Espinosa, C.; Fofanov, O.; Tøndel, C.; Chou, M.Z.; De Anda, C.S.; Kim, J.Y.; Li, D.; Sabato, P.; et al. Pharmacokinetics and Safety of Single-Dose Tedizolid Phosphate in Children 2 to <12 Years of Age. *Pediatr Infect Dis J* **2021**, *40*, 317–323, doi:10.1097/INF.0000000000003030.
42. Food and Drug Administration Ceftazidime-Avibactam New Drug Application Approval Letter 2015.
43. Food and Drug Administration Ceftazidime - Avibactam Supplemental New Drug Application Approval Letter 2018.
44. Food and Drug Administration Ceftazidime - Avibactam Supplemental New Drug Application 3 Months to Less Than 18 Years of Age Approval Letter 2019.
45. Food and Drug Administration Ceftazidime - Avibactam Supplemental New Drug Application 3 Months to Less Than 18 Years of Age HABP/VABP Approval Letter 2022.
46. Food and Drug Administration Ceftazidime - Avibactam Supplemental New Drug Application Birth to Less Than 3 Months of Age Approval Letter 2024.
47. Pfizer Evaluation of Safety, Pharmacokinetics and Efficacy of Ceftazidime and Avibactam (CAZ-AVI) Compared With Cefepime in Children From 3 Months to Less Than 18 Years of Age With Complicated Urinary Tract Infections (cUTIs) Available online: https://clinicaltrials.gov/study/NCT02497781?cond=NCT02497781&rank=1 (accessed on 23 July 2024).
48. Pfizer Evaluation of Safety,Pharmacokinetics and Efficacy of CAZ-AVI With Metronidazole in Children Aged 3 Months to 18 Years Old With Complicated Intra-Abdominal Infections (cIAIs) Available online: https://clinicaltrials.gov/study/NCT02475733?cond=NCT02475733&rank=1 (accessed on 23 July 2024).
49. Pfizer Evaluation of Pharmacokinetics, Safety, and Tolerability of Ceftazidime-Avibactam in Neonates and Infants. (NOOR) Available online: https://clinicaltrials.gov/study/NCT04126031?cond=NCT04126031&rank=1 (accessed on 23 July 2024).
50. Pfizer Single-Dose PK Study of Ceftazidime-Avibactam In Hospitalized Children Receiving Systemic Antibiotics for Nosocomial Pneumonia Available online: https://clinicaltrials.gov/study/NCT04040621?cond=NCT04040621&rank=1 (accessed on 23 July 2024).
51. Bradley, J.S.; Roilides, E.; Broadhurst, H.; Cheng, K.; Huang, L.-M.; MasCasullo, V.; Newell, P.; Stone, G.G.; Tawadrous, M.; Wajsbrot, D.; et al. Safety and Efficacy of Ceftazidime-Avibactam in the Treatment of Children ≥3 Months to <18 Years With Complicated Urinary Tract Infection: Results from a Phase 2 Randomized, Controlled Trial. *Pediatr Infect Dis J* **2019**, *38*, 920–928, doi:10.1097/INF.0000000000002395.
52. Bradley, J.S.; Broadhurst, H.; Cheng, K.; Mendez, M.; Newell, P.; Prchlik, M.; Stone, G.G.; Talley, A.K.; Tawadrous, M.; Wajsbrot, D.; et al. Safety and Efficacy of Ceftazidime-Avibactam Plus Metronidazole in the Treatment of Children ≥3 Months to <18 Years With Complicated Intra-Abdominal Infection: Results From a Phase 2, Randomized, Controlled Trial. *Pediatr Infect Dis J* **2019**, *38*, 816–824, doi:10.1097/INF.0000000000002392.
53. Food and Drug Administration Delafloxacin New Drug Application Approval Letter 2017.
54. Food and Drug Administration Delafloxacin Supplemental New Drug Application Approval Letter 2019.
55. Melinta Therapeutics, Inc. A Study Comparing the Bioavailability of a Taste-Masked Delafloxacin Powder for Oral Suspension With the Delafloxacin Tablet in Healthy Adults Available online: https://clinicaltrials.gov/study/NCT06612255 (accessed on 21 February 2025).
56. Food and Drug Administration Meropenem-Vaborbactam New Drug Application Approval Letter 2017.
57. Rempex (a wholly owned subsidiary of Melinta Therapeutics, LLC) Dose-Finding, Pharmacokinetics, and Safety of VABOMERE in Pediatric Subjects With Bacterial Infections (TANGOKIDS) Available online: https://clinicaltrials.gov/study/NCT02687906?cond=NCT02687906&rank=1 (accessed on 23 July 2024).

58. Rempex (a wholly owned subsidiary of Melinta Therapeutics, LLC) A Study to Evaluate the Safety, Tolerability, and Pharmacokinetics of Meropenem-Vaborbactam in Children With Complicated Urinary Tract Infection, Including Acute Pyelonephritis Available online: https://clinicaltrials.gov/study/NCT06672978 (accessed on 21 February 2025).
59. Food and Drug Administration Eravacycline New Drug Application Approval Letter 2018.
60. Tetraphase Pharmaceuticals, Inc. A Safety and PK Study of IV Eravacycline Available online: https://clinicaltrials.gov/study/NCT03696550?cond=NCT03696550&rank=1 (accessed on 23 July 2024).
61. Innoviva Specialty Therapeutics A Study to Evaluate the Safety and Tolerability of Eravacycline in Children Aged 8 to 17 With Complicated Intra-Abdominal Infections (cIAI) Available online: https://clinicaltrials.gov/study/NCT06794541 (accessed on 21 February 2025).
62. Food and Drug Administration Omadacycline New Drug Application Approval Letter 2018.
63. Paratek Pharmaceuticals Inc Study to Evaluate the PK of IV and PO Omadacycline in Children and Adolescents With Suspected or Confirmed Bacterial Infections Available online: https://clinicaltrials.gov/study/NCT05217537?cond=NCT05217537&rank=1 (accessed on 23 July 2024).
64. Food and Drug Administration Plazomicin New Drug Application Approval Letter 2018.
65. Food and Drug Administration Cefiderocol New Drug Application Approval Letter 2019.
66. Food and Drug Administration Cefiderocol Supplemental New Drug Application Approval Letter 2020.
67. Shionogi Inc. A Study to Assess the Safety, Tolerability, and Pharmacokinetics of Cefiderocol in Hospitalized Pediatric Participants Available online: https://clinicaltrials.gov/study/NCT04215991?cond=NCT04215991&rank=1 (accessed on 23 July 2024).
68. Shionogi Inc. A Study to Assess the Safety, Tolerability, and Pharmacokinetics of Cefiderocol in Hospitalized Neonates and Infants Available online: https://clinicaltrials.gov/study/NCT06086626?cond=NCT06086626&rank=1 (accessed on 23 July 2024).
69. Food and Drug Administration Imipenem-Relebactam New Drug Application Approval Letter 2019.
70. Merck Sharp & Dohme LLC A Pharmacokinetics Study of MK-7655A in Pediatric Participants With Gram-Negative Infections (MK-7655A-020) Available online: https://clinicaltrials.gov/study/NCT03230916?cond=NCT03230916&rank=1 (accessed on 23 July 2024).
71. Merck Sharp & Dohme LLC Safety, Tolerability, Efficacy and Pharmacokinetics of Imipenem/Cilastatin/Relebactam (MK-7655A) in Pediatric Participants With Gram-Negative Bacterial Infection (MK-7655A-021) Available online: https://clinicaltrials.gov/study/NCT03969901 (accessed on 21 February 2025).
72. Bradley, J.S.; Makieieva, N.; Tøndel, C.; Roilides, E.; Kelly, M.S.; Patel, M.; Vaddady, P.; Maniar, A.; Zhang, Y.; Paschke, A.; et al. Pharmacokinetics, Safety, and Tolerability of Imipenem/Cilastatin/Relebactam in Children with Confirmed or Suspected Gram-Negative Bacterial Infections: A Phase 1b, Open-Label, Single-Dose Clinical Trial. *J Clin Pharmacol* **2023**, *63*, 1387–1397, doi:10.1002/jcph.2334.
73. Cox, E. Lefamulin New Drug Application Approval Letter 2019.
74. Food and Drug Administration Sulbactam-Durlobactam New Drug Application Approval Letter 2023.
75. Innoviva Specialty Therapeutics A Multicenter, Open-Label, Phase 1b Study to Assess the Pharmacokinetics, Safety, and Tolerability of Sulbactam-Durlobactam in Hospitalized Pediatric Patients From Birth to <18 Years Who Are Receiving Systemic Antibiotic Therapy for Suspected or Confirmed Acinetobacter Baumannii-Calcoaceticus Complex Infection Available online: https://clinicaltrials.gov/study/NCT06801223 (accessed on 21 February 2025).
76. Food and Drug Administration Cefepime-Enmetazobactam New Drug Application Approval Letter 2024.
77. Allecra A Study to Investigate PK, Safety, Tolerability of Cefepime-Enmetazobactam in Pediatric Participants With cUTI Available online: https://clinicaltrials.gov/study/NCT05826990?cond=NCT05826990&rank=1 (accessed on 23 July 2024).
78. Food and Drug Administration Ceftobiprole New Drug Application Approval Letter 2024.
79. Cumberland Pharmaceuticals VIBATIV® (Telavancin). Package Insert. Available online: https://www.accessdata.fda.gov/drugsatfda_docs/label/2020/022110s016lbl.pdf (accessed on 27 March 2025).
80. BioSpace Theravance, Inc. Reports Fourth Quarter and Full Year 2009 Financial Results Available online: https://www.biospace.com/article/theravance-inc-reports-fourth-quarter-and-full-year-2009-financial-results-/ (accessed on 12 July 2024).
81. Cumberland Pharmaceuticals, Inc Cumberland Pharmaceuticals Reports 11.6% Fourth Quarter 2024 Revenue Growth Available online: https://www.prnewswire.com/news-releases/cumberland-pharmaceuticals-reports-11-6-fourth-quarter-2024-revenue-growth-302391883.html (accessed on 3 April 2025).
82. Allergan USA, Inc TEFLARO® (Ceftaroline Fosamil). Package Insert. Available online: https://www.accessdata.fda.gov/drugsatfda_docs/label/2021/200327Orig1s028lbl.pdf (accessed on 27 March 2025).
83. Teva Pharmaceutical Industries Ltd Teva Delivers Second Consecutive Year of Growth; Announces Strong Financial Results in Fourth Quarter and Full Year 2024, Led by Generics Performance and Innovative Portfolio Growth Available online: https://ir.tevapharm.com/news-and-events/press-releases/press-release-details/2025/Teva-Delivers-Second-Consecutive-Year-of-Growth-Announces-Strong-Financial-Results-in-Fourth-Quarter-and-Full-Year-2024-Led-by-Generics-Performance-and-Innovative-Portfolio-Growth/default.aspx (accessed on 3 April 2025).
84. Merck & Co., Inc ZERBAXA® (Ceftolozane and Tazobactam). Package Insert Available online: https://www.accessdata.fda.gov/drugsatfda_docs/label/2022/206829s011s012lbl.pdf (accessed on 3 April 2025).
85. Business Wire Cubist Reports Fourth Quarter and Full Year 2013 Financial Results; Provides 2014 Revenue Guidance Available online: https://www.businesswire.com/news/home/20140123006255/en/Cubist-Reports-Fourth-Quarter-and-Full-Year-2013-Financial-Results-Provides-2014-Revenue-Guidance (accessed on 19 February 2025).
86. Merck Sharp & Dohme Merck Announces Fourth-Quarter and Full-Year 2024 Financial Results Available online: https://www.merck.com/news/merck-announces-fourth-quarter-and-full-year-2024-financial-results/ (accessed on 3 April 2025).
87. AbbVie Inc. Dalvance (Dalbavancin). Package Insert Available online: https://www.accessdata.fda.gov/drugsatfda_docs/label/2025/021883Orig1s012lbl.pdf (accessed on 27 March 2025).
88. PR Newswire Actavis Net Revenue Increases 59% to $2.779 Billion in Fourth Quarter 2013; Non-GAAP EPS Increases 99% to $3.17 Available online: https://www.prnewswire.com/news-releases/actavis-net-revenue-increases-59-to-2779-billion-in-fourth-quarter-2013-non-gaap-eps-increases-99-to-317-246309901.html (accessed on 3 April 2025).

89. PR Newswire Actavis Successfully Completes Durata Therapeutics, Inc. Tender Offer Available online: https://www.prnewswire.com/news-releases/actavis-successfully-completes-durata-therapeutics-inc-tender-offer-282914211.html (accessed on 19 February 2025).
90. AbbVie AbbVie Reports Full-Year and Fourth-Quarter 2024 Financial Results | AbbVie Available online: https://investors.abbvie.com/news-releases/news-release-details/abbvie-reports-full-year-and-fourth-quarter-2024-financial (accessed on 3 April 2025).
91. Melinta Therapeutics, LLC ORBACTIV® (Oritavancin). Package Insert. Available online: https://www.accessdata.fda.gov/drugsatfda_docs/label/2022/206334s007lbl.pdf (accessed on 27 March 2025).
92. BioSpace The Medicines Company Reports Fourth Quarter And Full Year 2013 Financial Results Available online: https://www.biospace.com/article/the-medicines-company-reports-fourth-quarter-and-full-year-2013-financial-results-/ (accessed on 12 July 2024).
93. Merck & Co., Inc SIVEXTRO (Tedizolid Phosphate). Package Insert. Available online: https://www.accessdata.fda.gov/drugsatfda_docs/label/2021/205435s014,205435s009lbl.pdf (accessed on 27 March 2025).
94. AbbVie Inc. AVYCAZ (Ceftazidime and Avibactam). Package Insert. Available online: https://www.accessdata.fda.gov/drugsatfda_docs/label/2025/206494s013lbl.pdf (accessed on 27 March 2025).
95. Wasserman, E. Forest Laboratories, Inc. Reports Fiscal Year Fourth Quarter 2014 | Fierce Pharma Available online: https://www.fiercepharma.com/pharma/forest-laboratories-inc-reports-fiscal-year-fourth-quarter-2014 (accessed on 12 July 2024).
96. Food and Drug Administration, J. Delfafloxacin New Drug Application Approval Letter 2017.
97. Melinta Therapeutics, Inc BAXDELA (Delafloxacin). Package Insert. Available online: https://www.accessdata.fda.gov/drugsatfda_docs/label/2019/208610s007,208611s006lbl.pdf (accessed on 27 March 2025).
98. macrotrends Melinta Therapeutics Revenue 2010-2019 | MLNTQ Available online: https://www.macrotrends.net/stocks/charts/MLNTQ/melinta-therapeutics/revenue (accessed on 19 February 2025).
99. Melinta Therapeutics, LLC VABOMERE® (Meropenem and Vaborbactam). Package Insert. Available online: https://www.accessdata.fda.gov/drugsatfda_docs/label/2024/209776s009lbl.pdf (accessed on 27 March 2025).
100. Zippia Tetraphase Pharmaceuticals Revenue Available online: https://www.zippia.com/tetraphase-pharmaceuticals-careers-11368/revenue/ (accessed on 12 July 2024).
101. Tetraphase Pharmaceuticals, Inc. XERAVA® (Eravacycline). Package Insert. Available online: https://www.accessdata.fda.gov/drugsatfda_docs/label/2024/211109s009lbl.pdf (accessed on 27 March 2025).
102. CompaniesMarketCap Revenue History for Paratek Pharmaceuticals from 2004 to 2023 Available online: https://companiesmarketcap.com/paratek-pharmaceuticals/revenue/ (accessed on 12 July 2024).
103. Paratek Pharmaceuticals, Inc. NUZYRA (Omadacycline). Package Insert. Available online: https://www.accessdata.fda.gov/drugsatfda_docs/label/2021/209816s011,209817s010lbl.pdf (accessed on 27 March 2025).
104. Business Insider Achaogen Reports Fourth Quarter and Full Year 2017 Financial Results and Provides Corporate Update | Markets Insider Available online: https://markets.businessinsider.com/news/stocks/achaogen-reports-fourth-quarter-and-full-year-2017-financial-results-and-provides-corporate-update-1017344343 (accessed on 12 July 2024).
105. PitchBook Cipla 2025 Company Profile: Stock Performance & Earnings | PitchBook Available online: https://pitchbook.com/profiles/company/62947-27 (accessed on 3 April 2025).
106. Cipla USA, Inc. ZEMDRI (Plazomicin) Available online: https://www.accessdata.fda.gov/drugsatfda_docs/label/2023/210303s007lbl.pdf (accessed on 27 March 2025).
107. CompaniesMarketCap. Revenue History for Shionogi from 2009 to 2024 Available online: https://companiesmarketcap.com/shionogi/revenue/ (accessed on 3 April 2025).
108. Shionogi Inc. FETROJA (Cefiderocol). Package Insert. Available online: https://www.accessdata.fda.gov/drugsatfda_docs/label/2021/209445s004lbl.pdf (accessed on 27 March 2025).
109. Merck & Co., Inc Merck Announces Fourth-Quarter and Full-Year 2018 Financial Results Available online: https://www.merck.com/news/merck-announces-fourth-quarter-and-full-year-2018-financial-results/ (accessed on 12 July 2024).
110. Merck & Co., Inc RECARBRIO™ (Imipenem, Cilastatin, and Relebactam). Package Insert. Available online: https://www.accessdata.fda.gov/drugsatfda_docs/label/2020/212819s002lbl.pdf (accessed on 27 March 2025).
111. CompaniesMarketCap Revenue History for Nabriva Therapeutics from 2014 to 2023 Available online: https://companiesmarketcap.com/nabriva-therapeutics/revenue/ (accessed on 12 July 2024).
112. Nabriva Therapeutics US, Inc. XENLETA (Lefamulin). Package Insert. Available online: https://www.accessdata.fda.gov/drugsatfda_docs/label/2021/211672s002,211673s002lbl.pdf (accessed on 27 March 2025).
113. Stock Analysis Innoviva (INVA) Revenue 2015-2024 Available online: https://stockanalysis.com/stocks/inva/revenue/ (accessed on 3 April 2025).
114. Investor Relations - Innoviva Completes Acquisition of Entasis Therapeutics - Innoviva Available online: https://investor.inva.com/news-releases/news-release-details/innoviva-completes-acquisition-entasis-therapeutics (accessed on 12 July 2024).
115. La Jolla Pharmaceutical Company XACDURO® (Sulbactam; Durlobactam). Package Insert. Available online: https://www.accessdata.fda.gov/drugsatfda_docs/label/2023/216974Orig1s000Correctedlbl.pdf (accessed on 27 March 2025).
116. BioSpace Antibiotics Upstart Allecra Nabs $24.7 Million Series B Financing Available online: https://www.biospace.com/antibiotics-upstart-allecra-nabs-24-7-million-series-b-financing (accessed on 19 February 2025).
117. Allecra Allecra - Home Available online: https://www.allecra.com/ (accessed on 19 February 2025).
118. Allecra Therapeutics EXBLIFEP® (Cefepime and Enmetazobactam). Package Insert. Available online: https://www.accessdata.fda.gov/drugsatfda_docs/label/2024/216165s000lbl.pdf (accessed on 27 March 2025).
119. Basilea Pharmaceutica International Ltd Basilea Reports Strong 2024 Full-Year Results with Significant Increase in Revenue, Profit and Operating Cash Flow Available online: https://www.basilea.com/?tx_news_pi1%5Baction%5D=detail&tx_news_pi1%5Bcontroller%5D=News&tx_news_pi1%5Bnews%5D=1536&type=1546938654&cHash=562fe0ccfb9e81382c12f40631b143c9 (accessed on 3 April 2025).
120. Basilea Pharmaceutica International Ltd ZEVTERA (Ceftobiprole Medocaril Sodium). Package Insert. Available online: https://www.accessdata.fda.gov/drugsatfda_docs/label/2024/218275s000lbl.pdf (accessed on 27 March 2025).
